# HE4 (WFDC2) Promotes Tumor Growth in Endometrial Cancer Cell Lines

**DOI:** 10.3390/ijms14036026

**Published:** 2013-03-15

**Authors:** Jinping Li, Haibin Chen, Andrea Mariani, Dong Chen, Edward Klatt, Karl Podratz, Ronny Drapkin, Russell Broaddus, Sean Dowdy, Shi-Wen Jiang

**Affiliations:** 1 Department of Biomedical Science, School of Medicine, Mercer University, Savannah, GA 31404, USA; E-Mails: li_j@mercer.edu (J.L.); klatt_ec@mercer.edu (E.K.); 2 Division of Gynecologic Surgery, Mayo Clinic, Rochester, MN 55905, USA; E-Mails: mariani.andrea@mayo.edu (A.M.); podratz.karl@mayo.edu (K.P.); dowdy.sean@mayo.edu (S.D.); 3 Curtis and Elizabeth Anderson Cancer Institute, Department of Laboratory Oncology Research, Memorial University Medical Center, Savannah, GA 31404, USA; 4 Department of Histology and Embryology, Shantou University Medical College, Shantou 515041, Guangdong, China; E-Mail: chenhb@stu.edu.cn; 5 Department of Pathology, Mayo Clinic Medical College, Rochester, MN 55905, USA; E-Mail: dong.chen@mayo.edu; 6 Department of Pathology, Harvard Medical School, Boston, MA 77030, USA; E-Mail: ronny_drapkin@dfci.harvard.edu; 7 Department of Pathology, the University of Texas MD Anderson Cancer Center, Houston, TX 77030, USA; E-Mail: rbroaddus@mdanderson.org

**Keywords:** endometrial cancer, human epididymis protein 4 (HE4), HE4 variant, proliferation, invasion, colony formation, tumorigenesis

## Abstract

HE4, also known as WFDC2, is a useful biomarker for ovarian cancer when either used alone or in combination with CA125. HE4 is also overexpressed in endometrial cancer (EC), but its function in cancer cells is not clear. In this study, we investigate the role of HE4 in EC progression. An HE4-overexpression system was established by cloning the HE4 prototypic mRNA variant (HE4-V0) into a eukaryotic expression vector. Following transfection, stable clones in two EC cell lines were selected. The effects of HE4 overexpression on cell growth and function were measured with the use of cell proliferation assay, matrigel invasion, and soft agar gel colony formation assays. HE4-induced cancer cell proliferation *in vivo* was examined in a mouse xenograft model. HE4 overexpression significantly enhanced EC cell proliferation, matrigel invasion, and colony formation in soft agar. Moreover, HE4 overexpression promoted tumor growth in the mouse xenograft model. HE4 overexpression enhanced several malignant phenotypes in cell culture and in a mouse model. These results are consistent with our previous observation that high levels of serum HE4 closely correlate with the stage, myometrial invasion and tumor size in patients with EC. This study provides evidence that HE4 overexpression directly impacts tumor progression in endometrial cancer.

## 1. Introduction

National Cancer Institute (NCI) statistics show that while there was an insignificant decline in the incidence of endometrial cancer (EC) from 1997 to 2006 (−0.4 annual percentage change), the mortality rate increased significantly in the same time period (+0.3 annual percentage change). These data seem to suggest a trend for an increasing frequency of more aggressive forms of EC in the United States [1], which underscores the need for a better understanding of the molecular mechanisms and pathways involved in EC pathogenesis.

We performed a proteomics analysis on EC patient samples and found that HE4, a putative protease inhibitor containing two WAP (Whey Acid Protein) domains [2–4], is significantly increased in the endometrioid subtype of EC. Tissue microarray and real-time PCR studies confirmed a high level of HE4 expression in both endometrioid and serous types of EC [5]. These results are consistent with those from other laboratories showing increased HE4 mRNA and protein expression in endometrial cancer tissues [6–10]. Our subsequent investigation demonstrated that while all the five HE4 mRNA variants (or isoforms) are detectable in various normal tissues with varying expression levels, their levels are significantly increased in EC compared to normal endometrium. Moreover, regression analysis indicated that the expression levels of HE4-V1, HE4-V3 and HE4-V4 closely correlate with patients’ disease-free survival [5]. In a recent study in breast cancer tissues, Kamei *et al.* demonstrated that, HE4 expression levels are associated with both the lymph node metastases and decreased disease-free survival [11]. Furthermore, we observed that increased HE4 serum levels are associated with poor prognostic factors such as higher disease stage and deep myometrial invasion [5]. Taken together, these findings suggest that the function of HE4 may be directly involved in carcinogenesis. Interestingly, chromosome 20q, the region harboring the genes for HE4 and several WAP domain factors, is known to be a hot spot for frequent genetic amplification in EC, providing a potential mechanism that might be responsible for increased HE4 expression [12,13].

HE4, also designated as WFDC2 (WAP four-disulfide core domain 2), is a secretory protein detectable in human serum [3,14,15]. Correspondent to its overexpression in cancers arising from ovarian and endometrial [6–8], breast [11], and lung [2,6] tissues, HE4 serum levels are elevated in these cancer patients [3,10,14,16–18]. Most previous HE4 studies have concentrated on the development and improvement of the serum detection assay, as well as the clinical application of the HE4 test for ovarian cancer diagnosis, prognosis, and triage of patients with pelvic masses [16–26] These studies have shown that serum HE4 levels, alone or in combination with additional tests such as CA125, provide superior specificity and sensitivity to CA125 alone, or with other biomarkers [27]. Despite this progress, the cellular function of HE4, specifically its potential role(s) in cancer development, has not been systematically investigated. It is noteworthy that accumulated data has implicated WAP domain family members in cancer pathogenesis. Elafin and SLPI (secreted leukocyte protease inhibitor, or anti-leukoproteinase 1) are the two best-studied WAP proteins known to possess anti-protease activities. Elafin is expressed in human squamous cell carcinoma of the lung, but not in normal bronchial epithelium [28]. The immunostaining score of elafin was shown to be positively correlated with the squamous cell differentiation in head and neck, as well as esophageal tumors [29]. Clauss *et al.* have also shown that Elafin is overexpressed in serous ovarian cancer and correlated with poor overall survival [30]. SLPI is upregulated in a highly metastatic breast cancer cell line [31]. Immunohistochemistry studies on primary cancer tissues indicated a close association between SLPI levels and lymph node involvement [31]. Moreover, stable transfection experiments demonstrated that overexpression of either mouse or human SLPI led to increased tumorigenicity and lung-colonization potential of a low grade breast cancer cell line [32]. The prototypic HE4 protein contains two WAP domains that share some similarities with those from other WAP family members [33]. The prototype HE4 is encoded by a HE4 mRNA variant (NM_006103.3, HE4-V0) that is found at the highest levels in benign endometrium and EC tissues [5]. In this study, we chose to concentrate on the characterization of the HE4 effect on the malignant phenotypes of EC and the *in vivo* development of EC in a mouse xenograft model.

## 2. Results

### 2.1. Overexpression of Human HE4 in Endometrial Cancer Cell Lines

**Figure 1:**
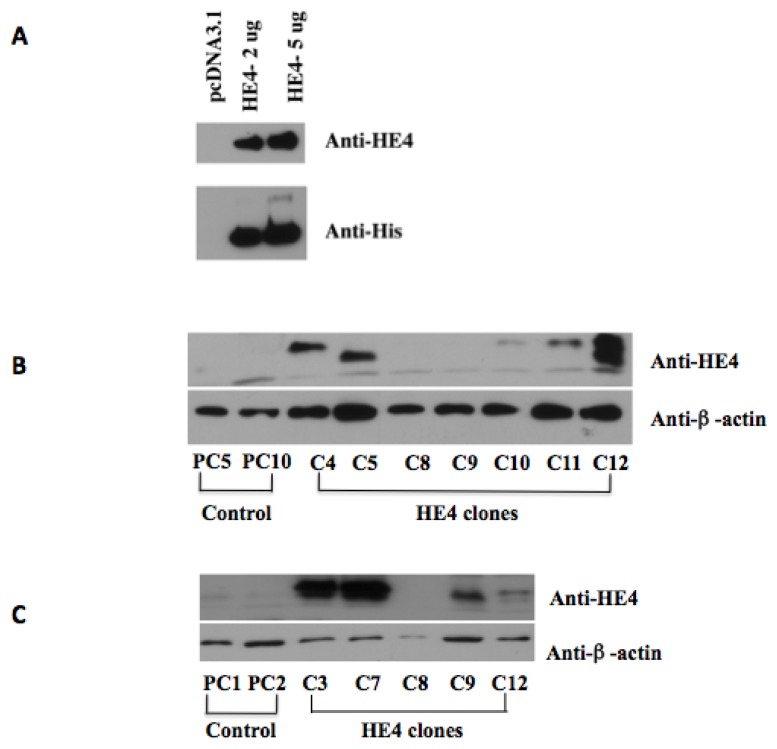
HE4 overexpression in EC cell lines. (**A**) Confirmation of HE4 overexpression following transient transfection. Ark2 cells were transfected with pcDNA 3.1-His-HE4 and the control plasmid pcDNA 3.1, respectively. Anti-HE4 and anti-His antibodies were used for Western blot analysis. A dramatically increased level of HE4 protein was detected in pcDNA 3.1-His-HE4 transfected cells. The same blot was detected with an antibody against β-actin and the result was used as protein loading control; (**B**,**C**) Confirmation of HE4 overexpression in stable clones. HEC-1B and Ark2 cells were transfected with pcDNA 3.1-His-HE4 and the control plasmid pcDNA 3.1. Stable clones were selected using geneticin and HE4 overexpression was examined by Western blot analysis. In HEC-1B cells (**B**), increased HE4 protein levels were found in HEC-1B-HE4-C4, -C5, -C11 and -C12 clones compared to the HEC-1B-PC5 and -PC10 control clones. In Ark2 cells (**C**), increased HE4 protein levels were detected in Ark2-HE4-C3, -C7, -C9, and -C12 clones over the Ark2-PC1 and -PC2 control clones.

Two endometrial cancer cell lines, HEC-1B containing relatively lower endogenous HE4 levels and Ark2 with relative higher endogenous HE4 levels (data not shown), were transfected with pcDNA 3.1-Myc-His-HE4 to achieve ectopic overexpression of HE4. Following transient transfection, HE4 protein levels were determined by real-time PCR and Western blot analysis (Figure 1A). The results confirmed that transfection with the HE4 construct was able to deliver HE4 overexpression in both cell lines. Stable transfection was subsequently performed and stable clones were selected using geneticin. HE4 levels in these clones were measured by real-time PCR (data not shown) and Western blot analysis. The four HEC-1B (HEC-1B-HE4-C4, -C5, -C11 and -C12) (Figure 1B) and four Ark2 (Ark2-HE4-C3, -C7, -C9 and -C12) (Figure 1C) clones with HE4 overexpression were selected. As previously reported by Drapkin, *et al*., in the HEC-1B-HE4-C12 clone, the HE4 antibody detected two bands with different migration rates, a phenomenon most likely caused by differential glycosylation modification of HE4 [4]. The HE4 positive clones and control clones (HEC-1B-PC5 and -PC10 for HEC-1B cells; Ark2-PC1 and -PC2 for Ark2 cells) were stored and subsequently used for studies on HE4 function.

### 2.2. HE4 Overexpression Stimulates Endometrial Cancer Cell Growth

**Figure 2:**
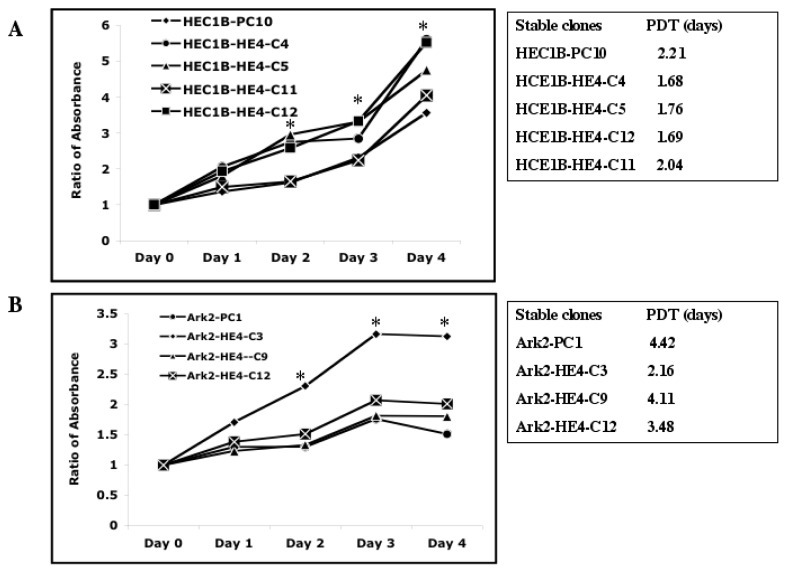
Cell proliferation assay in EC cells. (**A**) HEC-1B HE4 stable transfection clones (HEC-1B-HE4 C4, -C5, -C11, -C12) and control (-PC10) clone were grown in 48-well plates. The absorbance values were expressed as a ratio of Day 1 to 4 *versus* day 0 (set as 1). Growth curves show the relative absorbance at different time points (left panel). The ratios were converted to a logarithmic scale and the cell population doubling time (PDT) was calculated using the linear regression model (right panel). The HE4-overexpressing clones (HEC-1B-HE4-C4, -C5 and -C12) grew significantly faster from day 2 to Day 4 (*p* < 0.05, marked by *) and had shorter PDT than the PC10 control cells. Proliferation assay was also performed in Ark2 cells (**B**). As shown in the growth curves, the HE4-overexpressing clone (Ark2-HE4-C3) grew faster than the PC-1 control cells (*p* < 0.05, marked by *).

Altered cell proliferation represents a malignant feature that potentially contributes to cancer progression. To ascertain the effect of HE4 upregulation on HEC-1B and Ark2 cellular proliferation, the tetrazolium colorimetric cell proliferation assay was performed and the growth curves were shown in Figure 2. Compared to control cells, HEC-1B cells (HEC-1B-HE4-C4, -C5 and -C12 clones) with the highest levels of HE4 grew fastest, while HEC-1B-HE4-C11 expressing limited or lower levels of HE4 exhibited no significant change in proliferation. The population doubling time decreased from 2.21 days in control cells to 1.68 days, 1.76 days and 1.69 days for the HEC-1B-HE4-C4, -C5 and -C12 clones, respectively (Figure 2). Interestingly, HEC-1B-HE4-C4 and -C5 clones contain comparable levels of HE4 protein, and the two clones showed similar cell growth rates, although the HE4 protein migration rate was different (Figure 1A), suggesting the possibility that cell proliferation may not be closely related to HE4 protein posttranslational modification-glycosylation. Similar results were observed in Ark2 cell lines (data not shown). Since no significant change in either basal or inducer (cycloheximide or camptothecin)-mediated cell apoptosis was observed in HE4-overexpressing cells (data not shown), HE4 effects on cell proliferation appeared to be mostly due to an effect on the cell cycle. The positive impact of HE4 on cell proliferation in cell culture points to a potential role of HE4 for *in vivo* endometrial cancer growth.

### 2.3. HE4 Overexpression Enhances Invasive Ability and Anchorage-Independent Growth in EC Cells

**Figure 3:**
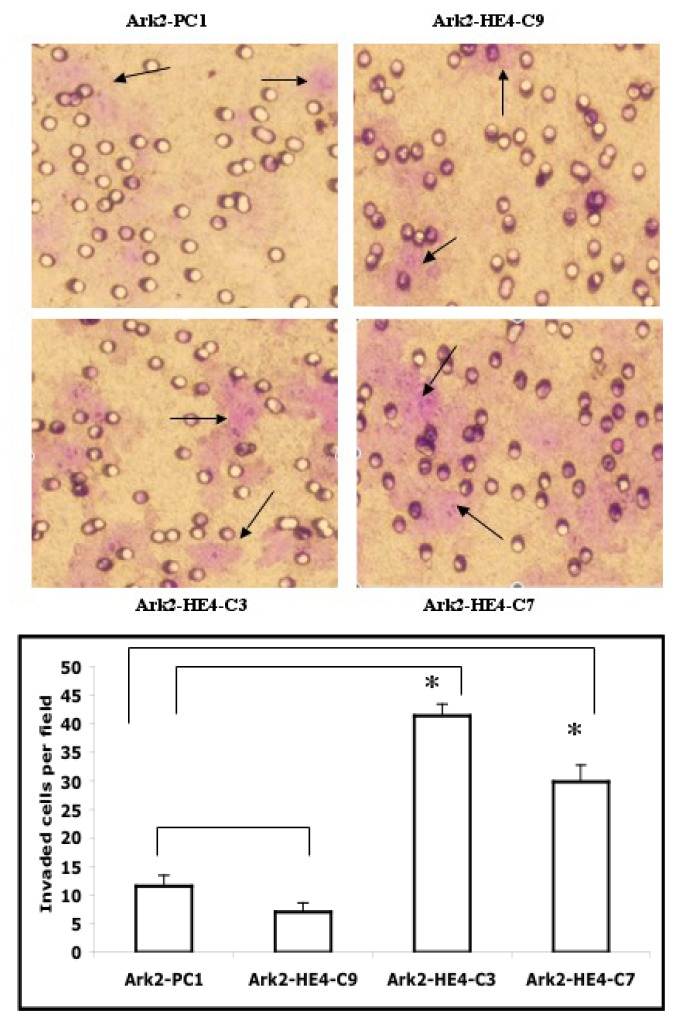
Effects of HE4 on EC cell invasion capability. HE4-overexpressing and control clones were subjected to matrigel invasion assay. The experiments were repeated three times, and one representative result is shown in the upper panel. Cell counting showed a significant increase of invasion activity (in light purple color, as indicated by arrows) in HE4-overexpressing Ark2-HE4-C3 and -C7 clones than in the Ark2-PC1 control clone (*p* < 0.05, marked by *, lower panel).

Matrigel invasion and anchorage-independent growth in soft agar are characteristics of malignant phenotypes found in various cancer cells. Figure 3 shows representative microscope fields for each of the stable clones and the cell counting results from the matrigel invasion assay. More cells from HE4-overexpressing clones (Ark2-HE4-C3 and -C7) invaded through the transwell compared to the Ark2-PC1 control clone. No significant change in the invasion ability was observed in the Ark2-HE4-C9 clone that expressed relatively low levels of HE4. A similar effect by HE4 overexpression was observed in HEC-1B cells (data not shown).

**Figure 4:**
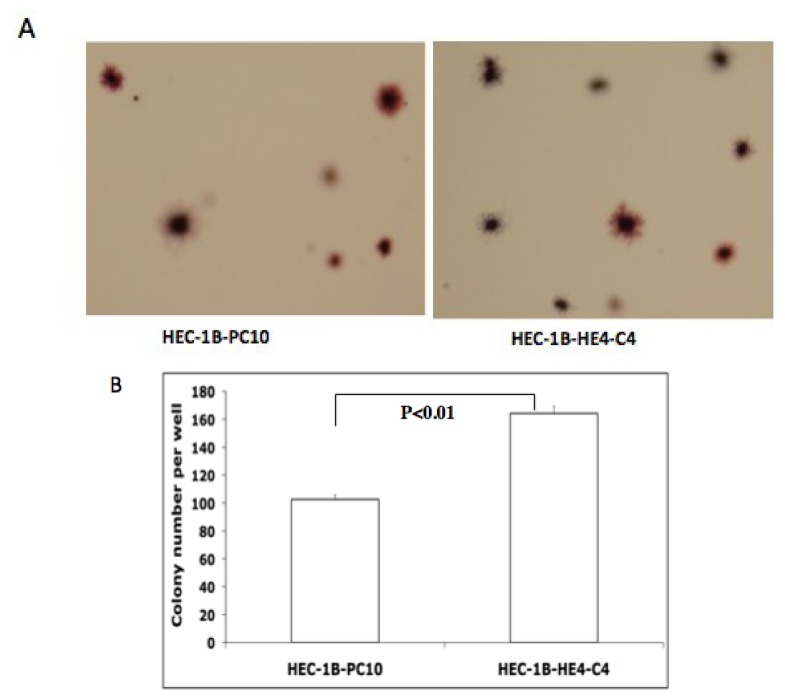
HE4 overexpression affects colony formation in soft agar. Control (HEC-1B-PC10) and HE4-overexpressing clones (HEC-1B-HE4-C4) were cultured in top soft agar for 22 days. (**A**) a representative result under low magnification, showing that colonies (stained in brown color) formed at the different depths of soft agar; (**B**) The average number of colonies and standard error for the two groups were calculated based on three independent experiments. The HE4-overexpressing cells formed significantly more colonies compared to control cells (*p* < 0.01).

The Transformation Detection Assay was performed according to the manufacturer’s protocols to examine anchorage-independent growth. As shown in Figure 4A, colonies were formed at different depths in the soft agar. The number of colonies from 10 randomly selected microscopic fields was counted and an average colony number for each group was shown in Figure 4B. HE4-overexpressing clones grew significantly more colonies than the control clones. Similar results were also obtained in the Ark2 EC cell line (data not shown). Overall, these results suggest that HE4 overexpression was able to confer a more malignant phenotype in EC cells.

### 2.4. HE4 Overexpression Promotes EC Tumor Growth *in Vivo*

**Figure 5:**
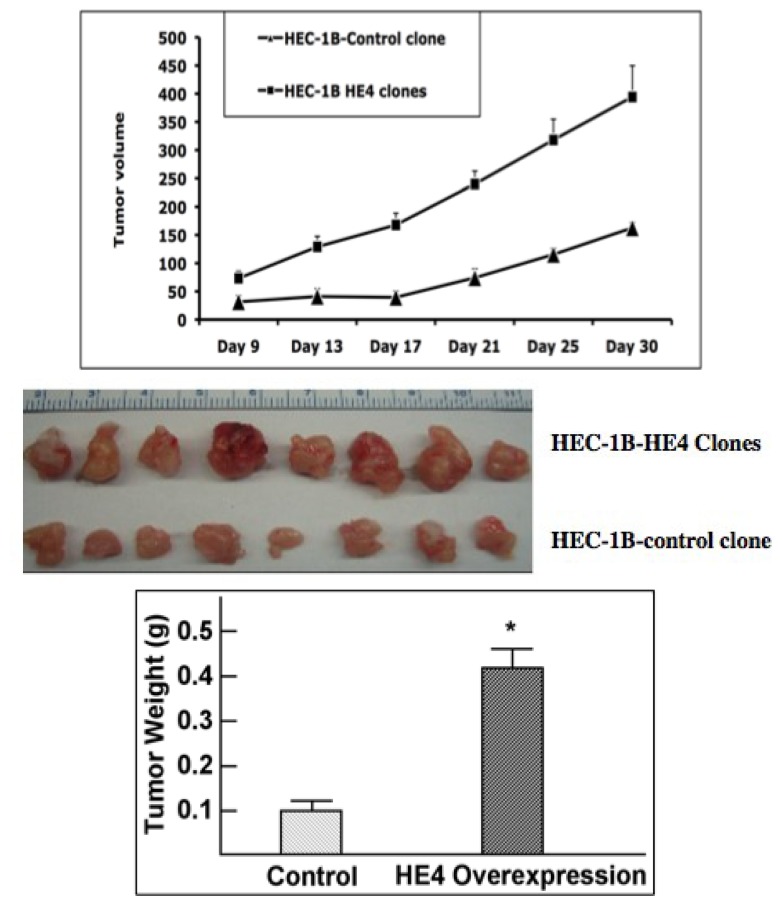
Effect of HE4-overexpression on xenograft tumor growth rate. HEC-1B HE4 overexpression and control clones were mixed, respectively, and inoculated subcutaneously into 8 SCID mice. Top panel: Growth curves showed that tumors formed by HE4-overexpressing cells grew faster than those by control cells. Middle panel: Tumors were dissected from mice on the 30th day. Bottom panel: Dissected tumors were weighed. The average weights and standard errors were calculated for each group. HE4-overexpressing cells grew tumors approximately three times larger than control cells (*p < 0.05).

Following the positive results from *in vitro* experiments, we further examined the effects of HE4 overexpression using a mouse xenograft model. The mixture of HE4-overexpressing clones and HEC-1B control clones were injected into SCID mice. Tumor growth curves were constructed by plotting tumor volumes against time (Figure 5, top panel). Tumors formed from HE4-overexpressing cells exhibited accelerated growth compared to the control cells. On the 30th day, the mice were sacrificed, and tumors were dissected and weighed (Figure 5, middle panel). Average tumor weights were compared between the two groups (Figure 5, lower panel). Consistent with the growth curves, the average final tumor weight from HE4-overexpressing cells was almost 3-fold heavier than that from the control cells. This effect is unlikely due to the artifact caused by clonal selection since a mixture of clones was used and a similar effect of HE4 overexpression was also observed when the Ark2 stable clones were injected to the SCID mice (data not shown).

### 2.5. Determination of HE4 mRNA and Protein Levels in EC Tumor Tissues

Total HE4 mRNA isolated from tumor tissues dissected from the xenograft model was measured. Real-time PCR results confirmed that tumors grown from HE4-overexpressing cells contained a significantly higher HE4 mRNA level than the tumors from control cells (data not shown). Immunohistochemistry using specific antibody against human HE4 confirmed, in accordance with the results from the mRNA measurement, that higher levels of HE4 were present in tumors derived from the HE4-overexpressing cells than those from control cells using Wilcoxon signed-rank test analysis (*Z* = −2.232, *p* = 0.026 (2-tailed), SPSS Software Version 19, IBM, New York, NY, USA). Representative images are shown in Figure S1. These results confirmed the continuing overexpression of HE4 mRNA and protein *in vivo* in the cells engineered for HE4 overexpression.

### 2.6. Tumors Overexpressing HE4 Have a Higher Number of Cells in the S-Phase

**Figure 6:**
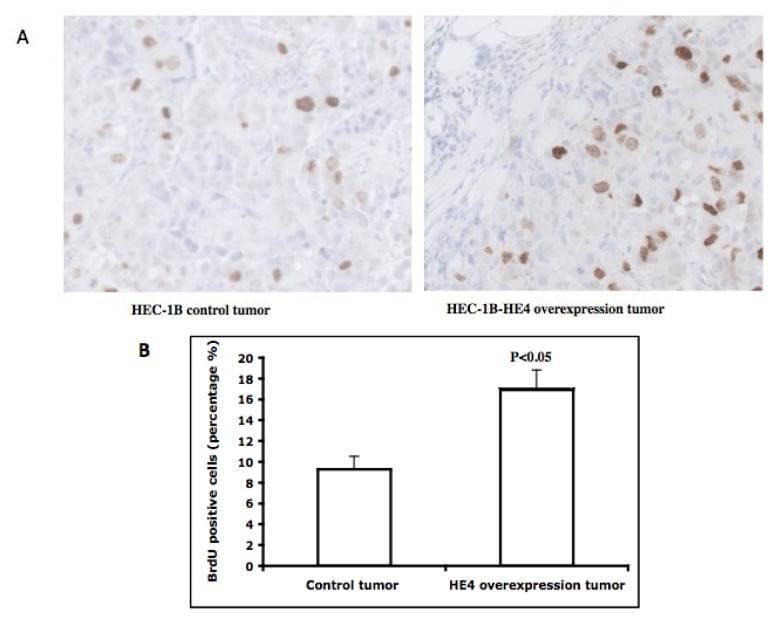
Tumor cell proliferation in xenografts. *In vivo* BrdU incorporation and immunostaining assays were performed in mice xenografts. (**A**) Representative staining results from the control and HE4-overexpressing groups; (**B**) The BrdU-positive (brown color) and negative nuclei were counted and the percentage of positive nuclei in total nuclei was calculated. Tumors from HE4-overexpressing cells contain a significantly higher percentage of BrdU-positive nuclei than tumors from control cells (*p* < 0.05).

We performed immunohistochemistry analyses on the tumor samples to examine if HE4 overexpression can impact tumor cell proliferation. Cells in S-phase were detected in the paraffin-embedded tumor tissues by immunostaining with BrdU antibodies. Representative fields of BrdU staining were shown in Figure 6A. The HE4-overexpressing tumors derived from HE4-overexpressing clones contained a higher average of BrdU-positive cells than those from the control clones (*p* < 0.05) (Figure 6B). This result confirmed the positive effects of HE4 overexpression on cell proliferation. Thus, the larger tumor mass following HE4 overexpression may be mostly accounted for by the enhancement of cell cycling.

## 3. Discussion

We have observed that five HE4 alternatively spliced variants are expressed in benign endometrial tissues, and that all of them are significantly increased in both type I and type II EC [5]. Moreover, HE4 serum levels are closely correlated with EC stage, myometrial invasion, and primary tumor diameter [5]. These findings suggest a potential role of HE4 in EC development. In this study, we employed cell cultures and animal models to characterize HE4 effects on EC malignant phenotypes, and we found that HE4 overexpression resulted in increased cancer cell proliferation, invasion capability, and anchorage independent growth. *In vivo* experiments indicated that HE4 overexpression promotes the EC xenograft growth in SCID mice. Thus, rather than being the byproducts of cancer cells and considered a surrogate marker for aggressive malignancies, HE4 may function as an oncogenic or tumor-promoting factor for EC development. To our knowledge, this is the first experimental evidence in support of a tumor-promoting role of HE4. We should point out that while our data reveal a potentially important function of HE4, the detailed molecular pathways/mechanisms regarding its upregulation and tumor-promoting actions during EC development remains unknown. Also, as an initial attempt to investigate HE4 function, our study has focused on the characterization of HE4-V0, the prototype HE4 variant expressed at the highest levels in human tissues. In normal endometrium, levels of HE4-V0 are 50-, 200-, 75-, and 143-fold higher than the levels of other four variants: HE4-V1, HE4-V2, HE4-V3, and HE4-V4, respectively [5] Follow-up investigations using a similar approach may help our understanding on the function of other HE4 variants in EC pathogenesis.

There appears to be overall agreement among our findings and those from our other two studies, ranging from cell culture, xenograft experiments, to clinical data regarding the HE4 expression and function. For example, cells expressing higher levels of HE4 exhibited faster proliferation than cells expressing lower levels of HE4. Correspondently, cells containing high levels of HE4 grew larger tumors than those with low HE4 expression in mouse xenografts. Clinical studies showed that high HE4 serum levels were correlated with later EC stage and larger primary tumor mass [34]. Similarly, we demonstrated that HE4 overexpression increases the ability of tumor cells to invade the matrigel, while higher HE4 serum levels were found to be associated with a greater tendency for myometrial invasion [34]. Interestingly, Kamei *et al.* performed HE4 immunohistochemistry on surgical specimens from breast cancer patients and found that lymph node metastases were associated with high HE4 expression [11]. Lymph node metastasis represents an important route for endometrial carcinomas, and lymph node involvement is a critical predictor of reduced survival. Unfortunately, our subcutaneous mouse xenograft model was not appropriate for investigating lymphatic spread, and future studies using a mouse uterine orthotopic model would be necessary to investigate this issue.

Studies from this and other laboratories indicate that HE4 mRNA and proteins were elevated in EC cells compared to normal endometrial glandular cells [5,6–8,10,35,36]. This provides the rationale for the use of an overexpression, rather than knockdown, approach to investigate HE4 function. Moreover, quantitative correlations between HE4 expression levels and functional data as observed in cell proliferation and cell invasion experiments suggest a dose-response effect for HE4 in EC cells. Our conclusion regarding the significant role of HE4 in EC cells is further strengthened by the consistency of the results from both the HEC-1B and Ark2 EC cell lines. It should be noted that cancer is a multistep process and different sets of genetic and epigenetic changes are involved in various steps [37,38]. Tumorigenesis, an early event associated with malignant transformation, and tumor growth promotion, a process more closely associated with tumor growth and metastasis, are thought to be two related—but largely different—processes [37]. Some tumorigenic factors contribute to malignant transformation, but not necessarily tumor growth and/or metastasis, and *vice versa*. Since our *in vitro* studies were all performed in cancer cell lines, the results are not informative regarding the role of HE4 in malignant transformation. Similarly, while the mouse xenograft model utilized in this investigation is reliable and convenient, the EC cells are readily tumorigenic in SCID mice and are therefore not suitable for determining if HE4 is tumorigenic *in vivo*. The HE4 transgenic mouse model may be useful for characterization of the carcinogenesis-specific role of HE4. Although we have used “tumor-promoting” to describe the overall effects of HE4 overexpression on EC cell proliferation and other malignant phenotypes, we recognize the non-specific and imprecise nature of this term. Without knowledge on the specific molecular mechanisms of HE4 action, it is not clear if HE4 affects tumor apoptosis, regulates the cell cycle, or encourages metastasis. Future studies concentrating on the HE4 protease inhibitor activity, the relationship between this activity and cell cycle regulation, and protein-protein interaction may help to elucidate the molecular pathways mediating the HE4 tumor-promoting activity.

The HE4 tumor-promoting activities appear similar to those found in other WAP proteins, namely elafin and SLPI. For those factors, the role in cancer development was thought to be related to their inhibitory effects on protease activity. However, additional functions, unrelated to anti-protease activity, have also been documented by some studies. For example, SLPI is capable of blocking activation of the NF-κB signaling pathway induced by lipopolysaccharide, which is unlikely to be related to protease inhibition [39]. Moreover, SLPI has anti-HIV activity, which is dependent not on its anti-protease activity but rather on its high affinity binding to the cell surface components of monocytes [40]. Thus, WAP domain factors may be capable of triggering signaling pathways [12] that regulate multiple downstream targets. Amino acid sequence-based bioinformatic analysis suggests that the WAP domains of HE4-V0 differ significantly from those of elafin and SLPI [33], and HE4-V0 is unlikely to carry any protease inhibitor activity. This observation suggests that the HE4 tumor-promoting function may not be mediated by a protease inhibition. Mutagenesis-directed sequence substitution and deletion studies are required to determine if the WAP domains are involved in the tumor-promoting activity of HE4.

Genetic and hormonal alterations are considered two major etiological factors for EC development. Many oncogenes achieve a high level of expression via genetic amplification. In humans, HE4, SLPI, and several other WAP members co-locate in 20q12-13 [12], a region frequently amplified in a variety of cancers [41]. Using comparative genomic hybridization (CGH), Micci *et al.* showed that gain of 20q was a frequent chromosomal abnormality in endometrial carcinomas [13]. These results suggest that somatic gain of genetic material may be a mechanism by which HE4 is expressed in high levels in endometrial cancer cells. Endometrium is also subject to tight control of both peptide and steroid hormone synthesis by changes in either hormonal levels or their receptors, and this may also contribute to the high HE4 levels as observed in endometrial cancer cells.

In summary, forced overexpression of HE4 promotes several malignant phenotypes including cell proliferation, cell invasion capability, anchorage independent growth, and increased tumor growth in endometrial cancer xenograft. Thus, the upregulated HE4 levels observed in primary endometrial cancer tissues may contribute to EC progression. Further studies are required to establish the molecular mechanisms responsible for the tumorigenic and tumor-promoting actions of HE4 in a variant-specific manner, and to understand the events leading to HE4 upregulation.

## 4. Experimental Section

### 4.1. Cell Culture

Ark2 and HEC-1B cells were purchased from ATCC and grown in RPMI1640 and DMEM/F12 medium, respectively. Both media were supplemented with fetal bovine serum (10% FBS) and antibiotics. Cells were grown to 50%–70% confluence before used for experiments.

### 4.2. Construction of Plasmid Vectors

The full-length coding region of human HE4 was generated by PCR using the following PCR primers: HE4 forward primer: 5′-CG GGA TCC ATG CCT GCT TGT CGC CTA GGC; HE4 reverse primer: 5′-GC GAA TTC G AAA TTG GGA GTG ACA CAG. The cDNA fragment was subcloned into the pcDNA3.1-His-myc-A vector (Invitrogen, Carlsbad, CA, USA). His-tag and myc-tag epitopes were incorporated to the *N*-terminus of HE4 peptide. Positive clones were confirmed by DNA sequencing at the Mayo Clinic Biochemistry Core Facility.

### 4.3. RNA Isolation and Reverse Transcription

Total RNA from EC and normal endometrium was isolated from 10-μm sections of frozen tissues using Trizol Reagent (Invitrogen, Carlsbad, CA, USA). Total RNA from EC cell lines was isolated according to the RNeasy Mini Kit (Qiagen, Valencia, CA, USA) protocol. All the RNA was treated with DNase I (30 Units/sample) to remove genomic DNA contamination. cDNA was synthesized with 1 μg of total RNA using the SuperScript™ kit (Invitrogen, Carlsbad, CA, USA) in 20-μL reactions. Reaction mixtures cDNA were diluted to 100 μL, and 1 to 3 μL was used for each real-time PCR reaction.

### 4.4. Real-Time PCR Analyses of mRNA Levels

Real-time PCR was performed using the Cyber Green PCR Master Mix (Invitrogen, Carlsbad, CA, USA) as previously described [42] using the following primers: HE4-Forward: 5′-ATA GCA CCA TGC CTG CTT GT; HE4-Reverse: 5′-TGC TCC TGT GCC TGA GAC TA. Housekeeping gene 36B4 was used as an internal reference: 36B4-Forward: 5′-ATG CAG CAG ATC CGC ATG T; 36B4-Reverse: 5′-TCA TGG TGT TCT TGC CCA TCA. To ensure the accuracy, the internal reference reaction was performed using the same sample as used for target gene. The results were standardized with the formula: CT_Δ_ = CT_Ref_ − CT_Target_. The results were further converted to folds of target gene over the reference gene (*F* = 2^CTΔ^).To compare the average values among different groups, ANOVA (analysis of variance) was performed to determine if there was an overall significant difference. For the data to satisfy the initial ANOVA criterion, individual comparisons were performed with the use of a *post hoc* Bonferroni *t* test with the assumptions of a two-tail distribution and two samples with equal variance using SPSS software.

### 4.5. *In Vitro* Proliferation Assay

The cell proliferation assay was carried out using the CellTiter 96 Aqueous Non-Radioactive Cell Proliferation Assay kit (Promega, Madison, WI, USA) according to the manufacturer’s protocol. A total of 2 × 10^4^ cells from the HE4-overexpressing clones and from control clones were grown in 48-well plates. Triplicate wells per clone were measured with absorbance at 490 nm (the absorbance at 620 nm was used as background). Cell numbers were evaluated every 24 h from the first day (day 0) to the fifth day (day 4). The data were expressed as a ratio of DNA content between day *X* and day 0. Ratios were converted to a logarithmic scale, and the cell population doubling times (PDT) was calculated using the linear-regression model. For each experiment, triplicate wells were seeded and examined. Three independent experiments were performed and the average results were presented.

### 4.6. Matrigel Invasion Assay

The experiments were carried out following established protocols [43]. A thin layer of matrigel (40 μL of 8 mg/mL stock solution; Becton-Dickinson Labware, Bedford, MA, USA) was overlaid on the upper surface of the 6.5 mm transwell chambers (8-μm pore size; CoStar, Corning, NY, USA). The matrigel was allowed to solidify by incubating the plates for 4 h at room temperature. Culture medium was added to the bottom chamber of the transwells. Stable clones for HE4 overexpression (Ark2-HE4-C3, -C7 and -C12) or for vector control (Ark2-PC1) were resuspended in 0.2% BSA/optimal medium at a concentration of 2 × 10^5^ cells/mL, and 5 × 10^4^ cells were added to the top well of the transwell chambers. Following 6 h of incubation, cells that had not invaded through the matrigel were removed from the upper surface using cotton swabs. Cells that invaded through the matrigel and reached the lower surface of the filters were fixed in methanol and stained with a 0.2% solution of crystal violet. Invasion was quantified by counting the cell number under a Nikon Diaphot microscope equipped with a 16-square reticle. The surface area of this grid was 1 mm^2^. Three separate fields were counted for each filter and the total numbers of cells were compared among experimental groups using the Student’s *t* test with the assumption of a two-tail distribution and two samples with equal variance. A difference of *p* < 0.05 was considered statistically significant.

### 4.7. Soft Agar Colony Formation Assay

The assay was performed with the use of a commercial kit (cat no. ECM570, Chemicon International a Serologicals Company, Emecula, CA, USA). Briefly, 2500 cells from the HE4 overexpressing clones (HEC-1B-HE4-C4) or control clones (HEC-1B-PC10) were mixed with 0.4% soft agar and plated on a layer of 0.8% of bottom agar in 12-well plates. 300 μL of complete medium was added on the top of agar. Cells were fed twice a week, and the plates were incubated for 22 days at 37 °C with 5% CO_2_ until colonies formed. Colonies were counted under the microscope. The experiment was repeated three times and the average numbers of colonies were calculated. The difference between the HE4-overexpressing clones and the control ones was analyzed with the use of the Student’s *t* test, applying an assumption of a two-tail distribution and two samples with equal variance. A difference of *p* < 0.05 was considered statistically significant.

### 4.8. Mouse Xenograft Model

Six-week old female severe combined immunodeficient (SCID)/beige mice were purchased from Charles River Laboratories (Wilmington, MA, USA) and housed in the institutional animal facilities. All animal experiments were performed under protocols approved by the Mayo Clinic Institutional Animal Care and Use Committee. Two million EC tumor cells with HE4 stable expression (mixture of HEC-1B-HE4-C4, -C5 and -C12 clones) or transfected with control vector (mixture of HEC-1B-PC5 and -PC10) were resuspended in 0.1 mL PBS and injected subcutaneously into the left flank of mice. Tumor growth was monitored every four days from the ninth day after tumor implantation by measuring the tumor axis. Tumor volumes were calculated by the formula *V* = 1/2*a* × *b*^2^, where *a* is the longest tumor axis, and *b* is the shortest tumor axis. On day 30, all mice were sacrificed by asphyxiation with CO**_2_**, and the tumors were dissected and weighed. Half of each tissue sample was fixed in 10% para-formaldehyde and preserved in paraffin blocks for further analysis. The other half were snap-frozen and stored at −80 °C for RNA isolation.

### 4.9. Detection of HE4 Expression in Tumor Tissues

Tissue sections were prepared as described above. An immunohistochemistry accessory kit (Bethyl laboratories, Inc., Montgomery, TX, USA) was used to examine expression levels of HE4 according to the manufacturer’s protocol. Briefly, after deparaffinization and rehydration, the slides were incubated in 3% hydrogen peroxide/methanol to quench the endogenous peroxidase. Slides were then incubated in hot Epitope Retrieval Buffer (provided in kit) for 20 min at 90–96 °C to recover epitopes. Non-specific reactions were blocked by incubating the sections with blocking reagent provided by the kit for 30 min at room temperature. 200 μL of human HE4 antibody (Signet Laboratory Inc., Dedham, MA, USA. Dilution 1:100) was applied to each slide and the slides were incubated at 4 °C overnight. Slides were subsequently incubated with secondary anti-rabbit IHC antibody as provided by the kit, and peroxidase activity was visualized with 3,3′-diaminobenzidine (DAB) substrate. Counterstaining was performed with hematoxylin. The stained sections from eight tumors in each group were reviewed and documented by a pathologist (D. Chen). HE4-positive staining was scored as 0 for negative, 1 for weak positive, 2 for positive, and 3 for strong positive in randomly selected high-power fields on the slide. The scores from the two groups were analyzed by the statistician using the Wilcoxon signed-rank test. *p* < 0.05 was considered statistically significant.

### 4.10. Tumor Cell Proliferation Assay *in Vivo*

Mice were injected with the Bromodeoxyuridine (BrdU) labeling reagent (Zymed Laboratories, South San Francisco, CA, USA; 1 mL/100 gram body weight) intraperitoneally 2 h before sacrifice. Tumor samples were dissected, fixed with 10% formalin overnight, and embedded in paraffin. Tissue sections were cut and mounted on slides. Staining was performed using the anti-BrdU kit according to the manufacture’s protocols (Zymed Laboratories, South San Francisco, CA, USA). Briefly, following deparaffinization in xylene and rehydration with a graded series of ethanol, slides were blocked with 3% hydrogen peroxide, digested with trypsin, and blocked with 1% horse serum to reduce the non-specific staining. Tissue sections were stained with a biotinylated mouse monoclonal anti-BrdU antibody (Zymed Laboratories, South San Francisco, CA, USA), and subsequently incubated with streptavidin peroxidase solution. The peroxidase activity was visualized with 3,3′-diaminobenzidine as substrate. Counterstaining was carried out with hematoxylin. The stained sections from 8 tumors for each group were reviewed. BrdU-positive nuclei were counted in 10 randomly selected high-power (40×) fields for each slide. The percentage of the BrdU-positive nuclei was calculated and average values were obtained for each group. The percentage difference between the HE4-overexpressing mice and the control mice was evaluated with the Student’s *t* test with the assumption of a two-tail distribution and two samples with equal variance. Statistically significant was considered when p value is less than 0.05.

## Supplementary Information
