# Supplementary material for: HE4 (WFDC2) Promotes Tumor Growth in Endometrial Cancer Cell Lines
Source: Int J Mol Sci. 2013 Mar 15;14(3):6026–43. doi: 10.3390/ijms14036026 (PMC3634435; doi:10.3390/ijms14036026)
Supplement: Supplementary file 1 [file ijms-14-06026-s001.docx]

Supplementary Information

**Figure S1.** Verification of HE4 expression levels in tumor tissues formed by HEC-1B cells. Mouse xenograft tumors were paraffin-fixed and immunohistochemistry was performed using HE4-specific antibody. Arrows indicate tumor cells. While weak or no staining signals were observed in the control tumor tissues, strong staining signals can be seen in tumor tissues formed by the HE4-overexpressing cells. Tissues were scored by a pathologist and the Wilcoxon signed-rank test showed a significant difference between the HE4 overexpressing and control groups (*p* = 0.026).

**
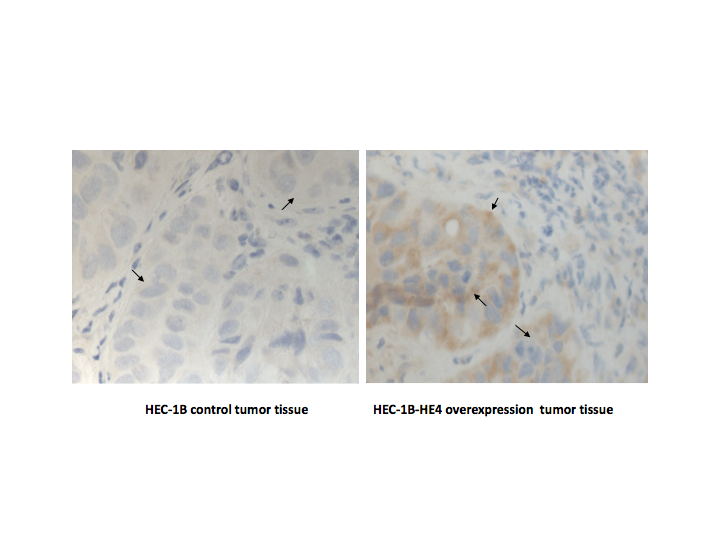
**

© 2013 by the authors; licensee MDPI, Basel, Switzerland. This article is an open access article distributed under the terms and conditions of the Creative Commons Attribution license (http://creativecommons.org/licenses/by/3.0/).
